# Notes and Notices

**Published:** 1889-12

**Authors:** 


### NOTES AND NOTICES.

Erratum.—In our September issue, page 389, line 19th from bottom, for *Organon* read *Organism.*

Guernsey's Diphtheria Card.—Dr. Wm. Jefferson Guernsey writes us that he has on hand a few of his Diphtheria cards, which he will mail to any one who will send postage and envelope for the purpose. These cards are well worth having.

Blind from a Snake Bite.—The New York *World* recently reported the case of a woman, living near Lafayette, Alabama, who was bitten on her hand by a rattlesnake; she became very ill for a few hours, then, seemingly entirely recovered from the effects of the bite, when she suddenly became entirely blind.

The annual competitive examination for Resident Physician and two Externes at the Children's Homœopathic Hospital, 914 N. Broad Street,

Philadelphia, will be held by the Medical Board during the latter part of March at the Institution.

A NEW HAHNEMANN CLUB has recently been organized at Terre Haute, Indiana, for the good purpose of studying the *Organon*. The Club will meet on the third Thursday of each month. Dr. Wilmot Moore was chosen President; Dr. W. R. Elder, Vice-President; Dr. W. H. Baker, Secretary; and Dr. A. L. Wilson Moore, Treasurer. The formation of so many Hahnemann Clubs throughout the country, is one of the most encouraging signs of the progress of Hahnemannian Homœopathy. Let the good work go on!

SURGICAL JOURNAL.—Of all the surgical journals we receive, we place the *Annals of Surgery* at the head. It is devoted exclusively to surgical work, and covers the entire field of surgery most exhaustively. It has an English as well as an American editor, with numerous collaborateurs; its data are collected wherever good surgical work is done. Subscription price $5 a year; publishers are Messrs. J. H. Chambers & Co., 914 Locust Street, St. Louis.

FOR SALE.—Very reasonably, a set of Lehrmann's, Jenichen's, and Fincke's high potencies; ranging from the 200th to the 100,000th. They include a list of four hundred remedies, in twelve hundred vials, contained in a black walnut chest. Six hundred vials contain liquids and six hundred pellets. For further particulars, address A. B., care of this journal.

ONE OF MANY.—We are constantly in receipt of letters from our subscribers telling us of the great help they receive from THE HOMŒOPATHIC PHYSICIAN. We quote from one recently received: "I will say that I owe more to THE HOMŒOPATHIC PHYSICIAN for introducing me to the true practice of medicine that to any other agency. The date of my subscribing to it was the beginning of a new era in my practice."

EPIDEMIC INFLUENZA.—In Russia, Germany, and France influenza has lately appeared in epidemic form. In Berlin Professor Von Leyden declares the disease has attacked a quarter of a million people, and has become a serious matter. Advices from Charkow declare an epidemic of typhus fever has followed closely upon the heels of this influenza. Some fear an epidemic of cholera may follow the influenza. The *cause of the influenza* is said to be "microbes."

REMOVALS—Dr. W. H. Baker, from Rochester, N. Y., to Terre Haute, Indiana; Dr. L. H. Lenke, from De Soto, Missouri, to the Homœopathic College at St. Louis; *The Medical Era*, from Chicago to Ann Arbor, Michigan; Richard J. Carter, M. D., from 54 W. 26th Street, to 18 W. 32d Street, New York City; Dr. E. H. Jewett, from 190 Erie Street, to 106 Euclid Ave., Cleveland, Ohio; Dr. F. E. Gladwin, from Chester, Penna., to the St. Louis Homœopathic College; Dr. C. M. Selfridge, from Santa Rosa, Cal., to Port Townsend, State of Washington; Dr. Ellis M. Santee, from Cortland N. Y., to the St Louis Homœopathic College; Dr. E. T. Balch, from Hoquiam, to South Bend, State of Washington; Mrs. M. J. Green, from Chillicothe to Kansas City, Missouri; W. H. Ross, M. D., from Louisville, to Skylight, Kentucky; Dr. Charles S. Mack, from Chicago to Ann Arbor, Michigan; Dr. H. P. Holmes, from Sycamore to Oak Park, Illinois; Dr. G. E. Gramm, from 1656 Vienna Street, to 1409 Hanover Street, Phila.; Dr. James T. Dicks, from 623 Russell Street, to 153 North Summer Street, Nashville, Tennessee; Dr. Charles W. Hakes, from New Milford, Pa., to Champaign, Illinois; Dr. A. T. Noe, from Nemaha City to Lincoln, Nebraska; Dr. S. Mills Fowler, from Dallas to Gainesville, Texas; Dr. J. A. Gill, from Seba, Arkansas, to Aurora, Missouri.

